# Three Dimensional Sculpturing of Vertical Nanowire Arrays by Conventional Photolithography

**DOI:** 10.1038/srep18886

**Published:** 2016-01-05

**Authors:** Run Shi, Chengzi Huang, Linfei Zhang, Abbas Amini, Kai Liu, Yuan Shi, Shuhan Bao, Ning Wang, Chun Cheng

**Affiliations:** 1 Department of Materials Science and Engineering and Shenzhen Key Laboratory of Nanoimprint Technology, South University of Science and Technology, Shenzhen 518055, China; 2 School of Computing, Engineering and Mathematics, University of Western Sydney, Kingswood, NSW 2751, Australia; 3 School of Materials Science and Engineering, Tsinghua University, Beijing 100084, China; 4 Department of Physics, Hong Kong University of Science and Technology, Hong Kong, China

## Abstract

Ordered nanoarchitectures have attracted an intense research interest recently because of their promising device applications. They are always fabricated by self-assembling building blocks such as nanowires, nanodots. This kind of bottom up approaches is limited in poor control over height, lateral resolution, aspect ratio, and patterning. Here, we break these limits and realize 3D sculpturing of vertical ZnO nanowire arrays (NAs) based on the conventional photolithography approach. These are achieved by immersing nanowire NAs in thick photoresist (PR) layers, which enable the cutting and patterning of ZnO NAs as well as the tailoring of NAs. Our strategy of 3D sculpturing of NAs promisingly paves the way for designing novel NAs-based nanoarchitectures.

Exercising rational control over nanostructures is necessary to tailor the functionalities and properties of various nanomaterials^1^,^2^,^3^,^4^. Ordered nanoarchitectures on substrate assembled by nanowire (NW) units are extremely desirable, in which anisotropic parameters, periodic structure and size may be tuned which could result in novel and promising properties for applications in optical^3^, thermal^5^, electric/electronic^6^ and energy^7^ related nanodevices. Various methods have been developed to fabricate patterned vertical nanowire arrays (NAs) in a large area on substrate; these methods are classified into three main categories: pre-patterned template methods^3^,^8^,^9^,^10^, post assembly methods^11^,^12^ and in-situ growth methods^4^,^13^,^14^. These existing approaches are limited to the two-dimensional patterning of NAs with a poor rough control of the NW length and/or a low patterning resolution owing to the unavoidable lateral growth or impurities induced growth. It is, therefore, desirable to realize large-scale sculpturing of vertical NWs on substrate though it remains one of the great challenges for nanotechnologists, preventing effective use of their promising properties and fabrication of practical devices. Recently, we developed a novel route to fabricate high-quality ZnO NAs with a controlled morphology and nanowire density directly from carbonized photoresist (PR) micro/nano patterns followed by chemical vapor deposition (CVD)^3^,^8^. Here, we achieved a further breakthrough for the three dimensional (3D) sculpturing of these NAs using a modified conventional photolithography technique. So far, we have realized a full and rational control of ZnO NAs CVD growth and structural tailoring.

## Results

**Figure 1:**
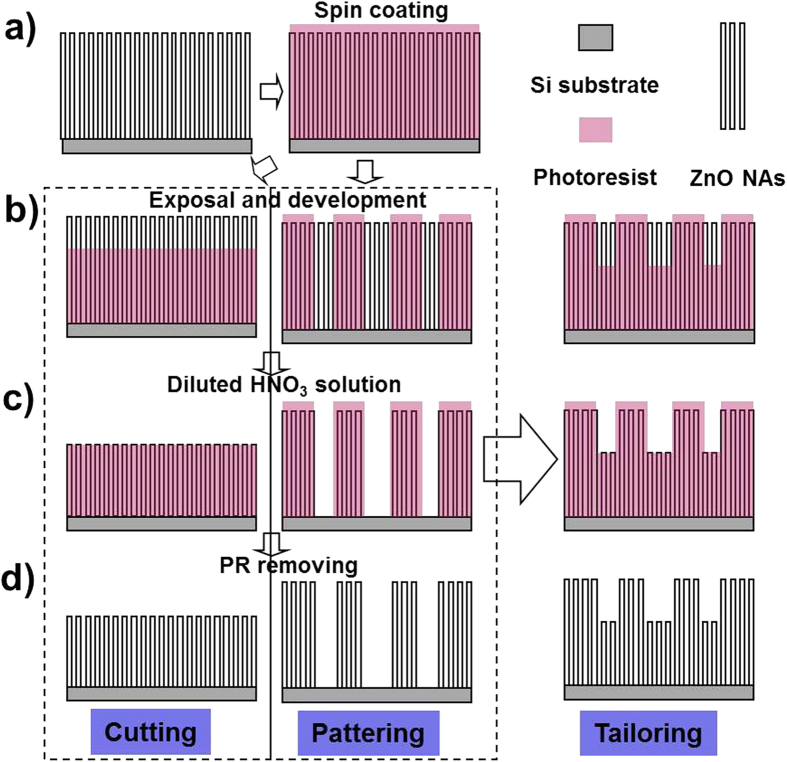
Fabrication process for the desired patterns of 3D sculpturing of nanoarrays (a) Spin coating; (b) Exposal and development; (c) Etching out the naked nanowires; (d) Photoresist removing.

Vertically aligned ZnO NAs grown on Si substrates by a CVD method^3^ was used for the 3D sculpturing of NAs based on a modified conventional photolithography technique. Figure 1 shows the process of two basic operations of the 3D sculpturing: cutting/shortening and patterning. Firstly, the ZnO NAs are wholly immersed in photo-resist (PR) by spin coating to form a ZnO NAs@PR matrix (Fig. 1a). This matrix is exposed to UV light and developed following a normal photolithography process (Fig. 1b). For the cutting of NAs, the exposure time is limited so that only a certain thickness of PR is removed along with the top part of NAs revealed after development. For the patterning of NAs, a mask with the desired pattern is used when taking exposure and a relatively long exposure time is required owing to the rather thick PR layer (See “Materials and Methods” Part for more details). Then, the matrix is dipped in a diluted HNO_3_ solution to etch out the naked part of NAs (Fig. 1c). The cutting and patterning of NAs are realized after removing the PR by washing the sample with acetone (Fig. 1d). Therefore, 3D sculpturing of vertical NAs can be easily achieved by the above combination of cutting and patterning.

**Figure 2:**
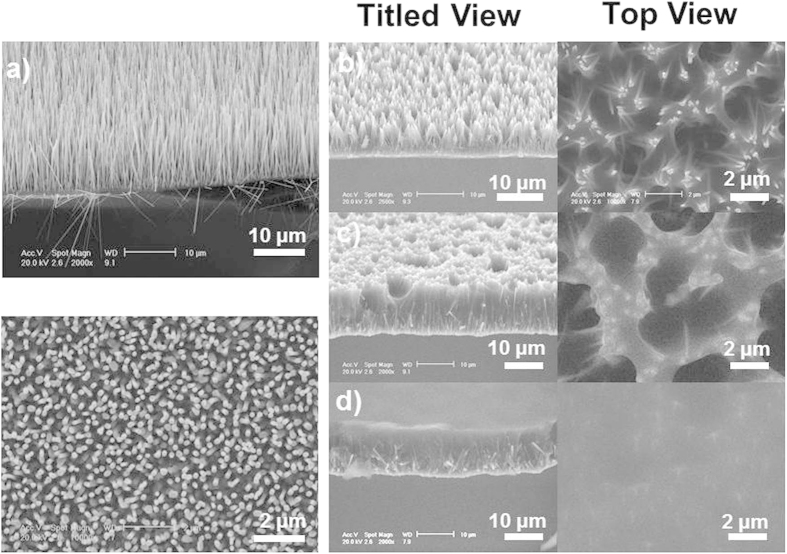
Titled view and top view (45°) of (a) original ZnO nanoarrays and those after (b) 1^st^, (c) 2^nd^, (d) 3^rd^ round of photoresist spin coating.

As shown in Fig. 1, the sculpturing process for NAs differs from that for thin films, in which PR is coated on the surface of substrates. A full filling of PR in ZnO NAs with a relatively flat surface is important for the sculpturing process. Figure 2a shows the SEM images of primary ZnO NAs recorded from top and titled views (45°). The height of the NAs is about 10 μm and the diameters of the nanowires range from 50 nm to 200 nm. After the first round of PR spin coating, ZnO NWs tend to aggregate into bundles, which consist of 10 ~ 20 NWs (Fig. 2b). After the second round of PR spin coating, the bundles connect with the PR and micro-sized pits form on the surface of ZnO NAs@PR matrix (Fig. 2c). Usually, all ZnO NAs are immersed in the PR with a flat surface after three standard rounds of PR spin coating (Fig. 2d).

**Figure 3:**
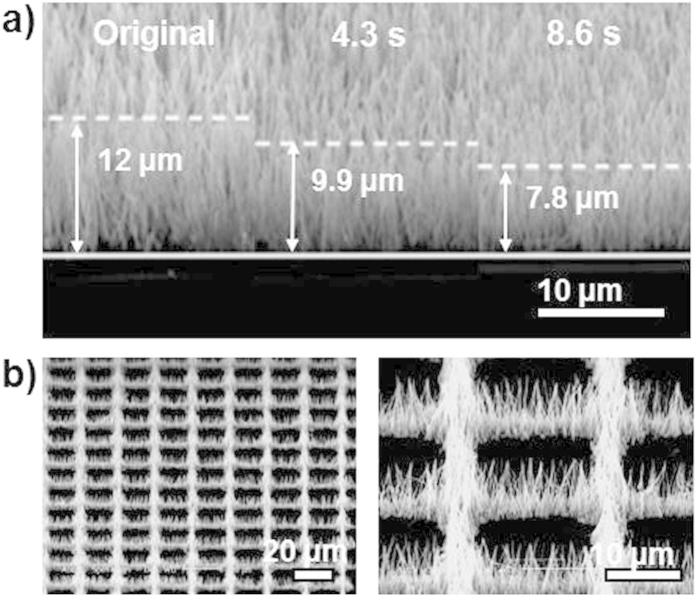
SEM images of (a) the results of ZnO nanoarrays with UV exposure times 0 s, 4.3 s and 8.6 s, (b) the square grid patterned ZnO nanoarrays and their enlarged image.

**Figure 4:**
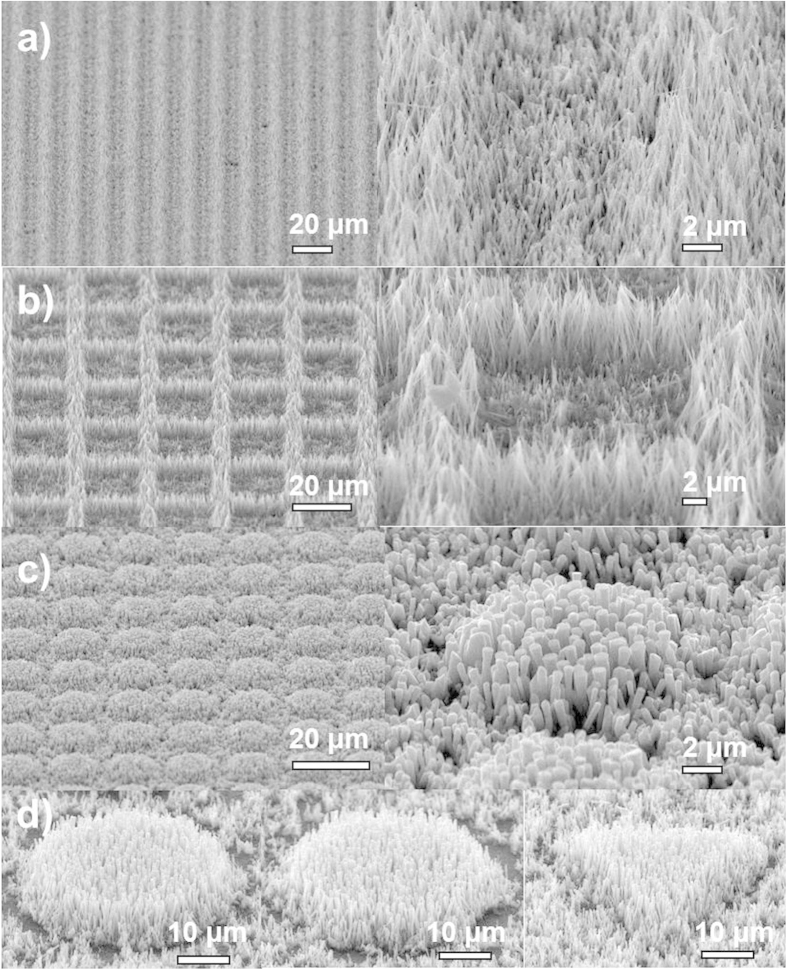
SEM images of sculpturing of ZnO nanoarrays with various patterns of (a) line arrays, (b) networks, and (c) disk arrays; (d) round, hexagonal and trigonal shaped ZnO nanoarrays; on the right side are the corresponding enlarged images.

**Figure 5:**
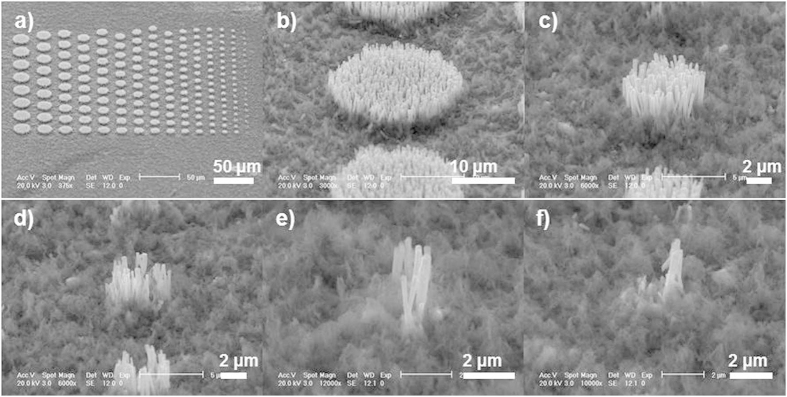
SEM images of ZnO nanoarrays sculptured with round patterns of different sizes.

Figure 3a shows the SEM images of height shortening results of ZnO NAs by the cutting process. The height of NAs decreases from 12 μm to 9.9 μm, about 2.1 μm shorter after being exposed for 4.3s. The shortening of NAs increases twofold, by about 4.2 μm, when the exposure time is increased by a factor of two to 8.6s. The height shortening of NAs increases linearly with exposure time. Therefore, the cutting process enables a precise control on the height of ZnO NAs, which is something hardly achieved by other self-assembly techniques on NAs^11^,^12^. Figure 3b shows SEM images of ZnO NAs with a square grid design achieved by the patterning process. It is found that the conventional photolithography technique can be well applied on NAs for 2D patterning and the resultant sample is similar to that achieved by patterned metal-catalysts/seeds guiding NAs growth^3^,^8^. Compared to other assembly techniques for patterned micro-nanostructures as mentioned in the introduction, our 3D sculpturing method shows remarkable advantages since cutting and patterning can be easily achieved simultaneously by conventional photolithography, and sculpturing of NAs can be expected. Figure 4 panels a-d illustrate several ZnO NAs tailored by our strategy, such as line arrays (Fig. 4a), networks (Fig. 4b), and disk arrays (Fig. 4c). The size, height, and shape of the ZnO NAs and their densities in one unit can be modified by photolithography conditions, such as masks and UV exposure time. Figure 4d shows round, hexagonal and trigonal shaped ZnO NAs, revealing an excellent shape sculpturing. Normally, the resolution for photolithography is about 2 μm. In order to find the smallest 2D feature size that can be achieved by our method, a mask with a pattern of a series of round disks, with sizes ranging from 1 μm to 20 μm, underwent the UV exposure process. Figure 5 shows that ZnO NAs with a disk diameter larger than 5 μm can still hold a round shape (Fig. 5b,c), however, those with a diameter smaller than 5 μm are fabricated into irregular shapes (Fig. 5d,e).

## Discussion

**Figure 6:**
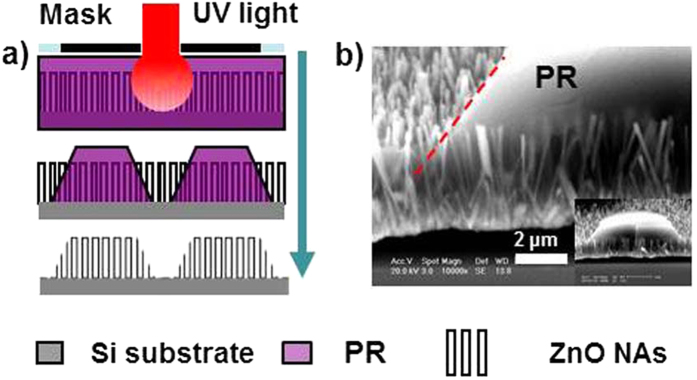
(**a**) Sketch map for the formation of the inclined planes at the pattern edge of the sculptured nanoarrays; (**b**) SEM image of nanoarrays@ photoresist after development treatment. The dashed line indicates the inclined profile of the photoresist edge.

As described above, the sculpturing process is based on a normal photolithography process except that the PR layer is rather thick and is replaced by a NAs@PR matrix layer. It was reported that vertically aligned NAs can effectively trap light and the gap between NWs helps the transmission of light^3^. These are the main reasons conventional photolithography can be applied to the NAs@PR matrix. In a normal photolithography process, a PR layer with a thickness of less than 2 μm is coated on the surface of the targeted substrates and the diffraction of light at the edge of a metal layer (usually Cr) on the mask is the main factor that degrades the photolithography resolution. To reduce this impact, a contact mode, in which the mask and substrate are closely attached, is the most common photolithography mode. Different from the above, in our case, the PR fills in the NAs and forms a relatively thick layer of up to 10 μm. Though the selected PR (AZ1518) is transparent to 400 nm UV light for exposure, the relatively long light transmission path will definitely reduce the resolution of the sculpturing owing to an enhanced diffraction of light. Furthermore, the aspect ratio and the diameter of NWs greatly affect the sculpturing results. When the light enters the NAs@PR matrix, the reflection and refraction across the interface of PR and NWs result in a diffused light dose distribution. As observed in Figs 2 and 3, NWs with a large aspect ratio tend to aggregate into bundles owing to capillary effect^15^ after being coated with PR and this significantly blocks the light transmission and enhances light scattering. NWs with a large diameter are rigid and hardly aggregate but they still contribute to light scattering. As a consequence, the pattern edge of the sculptured NAs is usually expected to possess inclined planes, which is confirmed by the observed results as shown in Figs 3 and 4. Figure 6a demonstrates the sketch map for the formation of inclined planes at the pattern edge of the sculptured NAs and Fig. 6b records the SEM images of the NAs@PR matrix after the development treatment. The edge of the patterned PR layer shows an inclined plane profile. This result indicates strong light scattering during the UV exposure process in the NAs@PR matrix, which is the main reason for the reduction of resolution of our sculpturing techniques. In order to achieve an acceptable performance, it is necessary to consider all related factors such as NW diameter, NW aspect ratio and the PR selection. Experimental results show that NWs with a diameter smaller than 200 nm and low aspect ratio of less than 20 are less affected by the light scattering when undergoing a sculpturing process (Fig. 5). A resolution of about 2.5 μm, close to that of conventional photolithography (2.0 μm), can be obtained. It is worthy to note that our sculpturing process is non-destructive on the final products. The used photoresist (AZ1518) is chemically inert, so that the naked part of ZnO NWs is etched away by HNO_3_ solution while the part of ZnO NWs immerged in the photoresist is well protected. In addition, solutions such as acetone, deionized water and treatment such as photoresist coating, low-temperature baking (120 °C) and UV light exposure definitely cannot introduce any structural and component changes on final ZnO NWs.

## Conclusions

In conclusion, we demonstrated a simple and effective method for 3D sculpturing of vertical ZnO NAs based on the conventional photolithography approach. Immersing ZnO NAs in thick PR layers enables the cutting and patterning of ZnO NAs as well as the tailoring of NAs. Combined with laser direct write lithography^16^, it is possible to extend the method for the sculpture of more complicated NAs-based nanoarchitectures. Furthermore, applying this method on nanoparticles @PR matrix will result in a novel approach for the fabrication of nanoarchitectures based on nanoparticles. With simplicity and excellent compatibility to complement metal oxide semiconductor processing, the as-developed method facilitates the fabrication of nanoarchitectures using NAs/nanoparticles as nano bricks in functional nanodevice applications such as resonators^17^, nanophotonics^18^, solar cells^19^, nanogenerators^7^, 3D FETs^20^,^21^, field emitters^22^,^23^, sensors^24^,^25^,^26^, and more.

## Methods

### Growth of ZnO NAs

ZnO NWs were synthesized using a vapor transport method reported previously^3^: An alumina boat containing 3 g of ZnO powder was placed in the center of a tube furnace. Si substrates with PR patterns were placed downstream for the nucleation and growth of ZnO NWs. The furnace was heated to 1300 °C and the temperature maintained for half an hour under vacuum conditions (~10^−2^ Torr). It was observed that ZnO NWs with a length of ~10 μm grew on the substrates when the temperature was about 700 ~ 900 °C.

### Sculpturing of ZnO NAs

As-grown ZnO NAs on Si substrates were coated with several layers of PR (AZ1518 photoresist) by spin coating at a speed of 4000 rpm for 30 s, and then treated by hard-baking at 120 °C for 60 s. The coating times of the PR layers varied with the length of NWs. In the following, we took ZnO NAs with a length of about 10 μm as examples and for this sample, the standard coating process was repeated for three times to form a NAs@PR matrix with NAs wholly immersed in PR. The as-formed ZnO NAs@PR matrix was positioned under photomasks using a mask aligner (ABM Inc.) and then exposed to 400 nm UV light. For the cutting of NAs, the UV exposure time was limited, so that only a certain thickness of PR was removed with the top part of NAs revealed after the developing step. For the patterning of NAs, a mask with the desired pattern was used and a relatively long UV exposure time of 30 s was required owing to the rather thick PR layer (10 μm). The nanowires in the UV exposed part of PR layer revealed after the developing step. Then, the matrix was dipped in diluted HNO_3_ solution (5% V/V) for 5 s to etch out the naked part of NAs (Fig. 1c, the same etching process and recipe for both the cutting and patterning of NAs). The cutting and patterning of NAs were realized, respectively, after removing the PR by washing the sample with acetone and deionized water (Fig. 1d). Therefore, 3D sculpturing of vertical NAs can be easily achieved by a combination of the above cutting and patterning process.

### Sample Characterizations

All the above samples were examined by a Philips scanning electron microscope (SEM, XL-30) and a JEOL high resolution transmission electron microscope (HRTEM, 2010F) equipped with an energy-dispersive X-ray spectrometer (EDX).

## Additional Information

**How to cite this article**: Shi, R. *et al.* Three Dimensional Sculpturing of Vertical Nanowire Arrays by Conventional Photolithography. *Sci. Rep.* **6**, 18886; doi: 10.1038/srep18886 (2016).
